# The Texas Medical College

**Published:** 1892-11

**Authors:** 


### THE TEXAS MEDICAL COLLEGE.

"Somebody has blundered." That the class of matriculates at the Texas Medical College at the opening of its second session should have been very little if any larger than it was the first session is a matter of surprise, and a mortifying disappointment to all friends of the institution. It is especially mortifying to those who have striven so faithfully to inaugurate a high grade school, and thus to advance the standard of medical education in Texas.

A magnificent building was constructed, and every department thoroughly equipped for teaching modern medicine. An able faculty was secured, and a high and thorough curriculum was arranged, extending over three years in graded courses. No longer should it be a reproach to Texas that with her princely school fund and her university, where all else is taught, her sons were compelled to go abroad for medical education. After ever so many obstacles overcome, at last the doors of a magnificently prepared medical college were thrown open and students were invited to come in. They didn't come.

The mistake was made by the legislature. They—and not the Regents—as should have been the case, fixed the fees and fixed them too high.

When we say fixed them too high, let it be understood that in this State a *diploma* is a *diploma*. There is no discrimination observed whatever, and practically, one diploma is as good as another. A man who can go to almost any Southern city and, after three months attendance on lectures, return to Texas and begin practice; or who can go, it may be, without intermission, from a three months' course at a winter school (which has cost him say $50 for tuition), right into a summer school, where, after three months more and another $50 fee, he can secure a diploma which will entitie him to recognition by the profession and all the rights and privileges of a legal practitioner of medicine in Texas, can hardly be expected, in these days, to sacrifice so much to patriotism as to attend three years at a home school and pay $140 tuition each year, *unless*, thereby, he could secure a diploma which would give him certain advantages over the cheaper sort; in brief, Texas cannot expect to keep her students at home so long as a diploma from a two year school and $50 fees is put upon the same footing before the law.

In these days many men secure a medical education as an investment, and engage in practice for a livelihood. With such men, a diploma that will pass "muster," one that will "do at all will do very well." We are not speaking of those who acquire a profound education because of the love of science, and for their own satisfaction, and because they have plenty of time and plenty of money. That a great many study medicine who ought never to attempt to do so, is evident; some will never make doctors; but how is it to be prevented? The fact is, that the majority of young men who elect medicine as a calling are poor and poorly educated; but they want to become doctors all the same, and so long as there are schools which adapt their requirements to the capacity and pecuniary circumstances of these students, so long as it is possible to get through in double quick time at a very light expense, it is folly to expect to build up such a school as is contemplated at Galveston.

And there is where the mistake was made. The professors being paid by the State, there is no necessity for a high fee, and especially as tuition in every other department in the University of Texas is *free*, except that of law—and there it is only nominal —the fee for tuition in medicine should have been placed even below that charged by any reputable school whose diploma will be recognized in Texas.

And, even then, it is highly problematical whether Texas students will elect to go through a three years' graded course with strict requirements both for matriculation and graduation, so long as the diploma they get will do no more for them than any other diploma. The State of Texas must do one of two things if she expects to build up her medical department: she must put the fees down to a merely nominal sum, or else protect the graduates from competition at the hands of those holding cheap diplomas from short term schools.

We can hardly hope, however, for a law especially to protect the graduates of our own school; it would be unreasonable to ask it. Every effort to secure legislation which will require something more than the mere possession of a diploma (from any source) as a requisite to the privilege of practicing medicine in Texas, has been laughed to scorn. And now, the senators who mocked those efforts are confronted with a problem hard to solve, and they must take their choice between keeping up a costly establishment and paying a teaching corps high salaries to lecture to empty benches—or they must offer Texas students other inducements, or protection from competition with cheaply made doctors. The first thing to be done, in our opinion, is to reduce the cost of lectures to the matriculation fee and anatomical fee only. If then our five hundred students still refuse to be highly and thoroughly educated, pass a law which will admit to practice in Texas no man whose diploma has been issued by a school requiring less than is required at Galveston.

At this writing, November 16th, there is in session in Louisville a convention of representatives of Southern Medical Colleges. It is thought they will enter into an agreement to require a three years' graded course as requisite to the degree. In case that is done, our course is very clear: the Regents of the Texas University should secure the passage of a bill which will recognize no other kind of a diploma.

The JOURNAL is advised that something of the kind is now beng considered. If such agreement is made, the medical societies of Texas will be called upon to endorse some such bill, to be presented to the legislature in January. At this writing, we are expecting a report from the Louisville meeting by our Associate Editor, Prof. Smith, who is present as representative of the Texas Medical College.

